# A Novel Technique for Quality Control of Microinjection Molding

**DOI:** 10.3390/mi17010074

**Published:** 2026-01-05

**Authors:** Abdel Naser Daoud, Atef M. Ghaleb, Zulfiqur Ali, Ali Abdelhafeez Hassan

**Affiliations:** 1 Department of Mechanical Engineering, College of Engineering & Advanced Computing, Alfaisal University, Riyadh 11533, Saudi Arabia; 2 Department of Industrial Engineering, College of Engineering & Advanced Computing, Alfaisal University, Riyadh 11533, Saudi Arabia; 3 University of Cumbria, Fusehill Campus, Carlisle CA1 2HH, UK; zulfiqur.ali@cumbria.ac.uk; 4 Institute of Engineering, Computing and Advanced Manufacturing, University of Cumbria, Barrow-in-Furness LA14 2PJ, UK; ali.hassan@cumbria.ac.uk

**Keywords:** microinjection molding, injection molding quality control, injection molding simulation, AI quality control, predictive control

## Abstract

In the microinjection molding process, continuous monitoring is important for optimization of the process and control. In microfluidic or lab-on-chip devices, defective microfeatures can compromise biological assays and diagnostic results, and therefore, the quality of these features is a critical issue. Microfeatures can be inspected using advanced inspection and microscopic techniques, but these are expensive, time-consuming, and difficult to use for full-scale production. We present here a new technique for quality control of microfeatures, which uses the filling of a controlled microcavity inside or outside the molded part as a quality control tool for filling microfeatures. Micro gaps (checkpoints) are used as an indicator of microfeature filling. Two micro gaps can be used for filling (checkpoints) as a Go/No-Go gauge.

## 1. Introduction

Conventional quality control methods for microfeatures are costly, complicated, and not suitable for mass production operations. There is a need for a simple, quick, cost-effective quality control measure for mass production.

**Figure 1:**
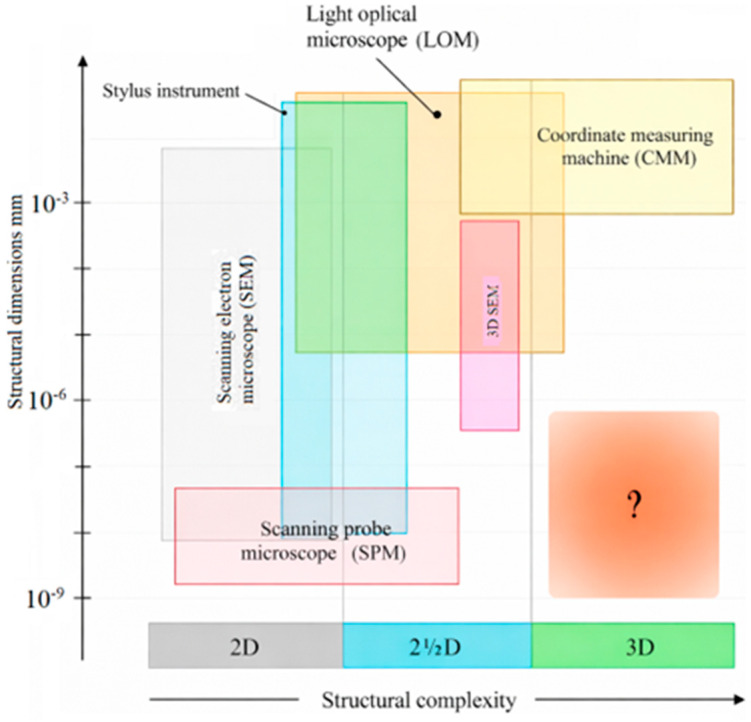
Categorization of equipment for dimensional micro metrology with respect to structural dimensions and structural complexity [6].

In general, microcomponents are characterized and inspected using advanced analytical and microscopic techniques. The equipment includes a coordinate measuring machine, a stylus profile meter, a scanning electron microscope (SEM), and optical microscopes [1]. The measurements are conducted with respect to micro and nano domains and are defined by characteristics such as roughness, geometry, and texture. Figure 1 provides a brief overview of the basic techniques and categorizes them with respect to structural dimensions and complexity.

Inspection techniques for measuring very small micro-molded parts require customized vices, tweezers, and fixtures. Only specialized inspection equipment is suitable for measuring at sub-micron tolerances, and cleanrooms with HEPA-filtered air and temperature control are required to ensure repeatable measurements [2]. Zhao et al. [3] used a cavity pressure sensor, proximity sensors, and a force transducer to monitor the microinjection molding filling behavior of polymer materials with different rheological properties. However, these types of sensors do not clearly indicate the polymer behavior inside the microcavity. Whiteside et al. [4] used injection pressure, cavity pressure transducers, a magnetostrictive sensor, and mold temperature thermocouples to evaluate the effect of molding conditions on the properties of the resultant component. Cheng et al. [5] designed an experimental setup using a He - Ne laser and a power meter together with a photodetector to measure the diffraction efficiency of gratings and the diffraction pattern of a laminar grating structure.

Marco et al. [7] suggest that improved quality products will force manufacturers to further increase the level of sophistication of finished parts, both qualitatively and quantitatively. The use of optical systems with digital image processing provides an effective solution for enhancing the reliability and repeatability of controls, reducing the time for component inspection, and providing information on the management of production processes. Daniel et al. [8] reported that the choice of an appropriate measurement setup for the quality inspection of metallic microcomponents will be influenced by, but is not limited to, specific parameters like cost, depth resolution, lateral resolution, feature dimensions, and defect size.

Recent developments in quality control for injection molding have focused on Industry 4.0 approaches with AI-driven quality control systems that detect defects in real time and ensure that only parts meeting exact specifications move forward in production [9].

Baldi et al. [10] investigated the process–property–structure relationship in miniaturized injection-molded components, demonstrating the importance of understanding material behavior at the microscale. Recent research by Chang et al. [11] developed an adaptive control system for injection molding using nozzle pressure sensors and tie-bar strain gauges to monitor melt quality in real time. The study established online quality characteristic values, including a viscosity index, peak pressure, and timing parameters for process optimization.

Bellantone et al. [12] proposed the use of surface parameters as quality indices for microinjection molding evaluation, demonstrating that surface roughness parameters such as Sa (arithmetic mean height) provide greater accuracy in part quality control than traditional dimensional measurements. Kun et al. [13] developed machine learning models using multilayer perceptron neural networks combined with quality indices extracted from pressure curves for automatic quality prediction of injection-molded parts.

Recent advances in microinjection molding quality control have emphasized the integration of artificial intelligence and machine learning techniques. Zhao et al. [14] defined intelligent injection molding as the integral application of sensing, optimization, and control procedures, reviewing methods for the detection of relevant physical variables and the optimization of process parameters. The study highlighted the potential for advanced sensing technologies to perceive physical fields throughout the molding process.

Recent developments in automated inspection systems include AI-driven quality control that can detect defects in real-time using computer vision and machine learning algorithms [15].

The integration of cavity pressure monitoring has emerged as a key technique for quality characterization, with researchers developing methods to correlate pressure curves with part quality metrics [16]. Advanced simulation techniques have become crucial for microinjection molding process validation, with recent studies demonstrating high precision validation methodologies [17].

The proliferation of point-of-care diagnostics and personalized medicine has created a significant demand for disposable, mass-produced microfluidic devices, often referred to as “lab-on-a-chip.” These devices perform complex biological analyses on a small plastic chip, relying on networks of precise microchannels, chambers, and microfeatures. The functional integrity of these microfeatures is paramount; incomplete filling or a defect in a critical microchannel during manufacturing can lead to catastrophic failure, resulting in erroneous diagnostic results or device malfunction. Given that point-of-care diagnostic devices have an impact on patient health, there is a particular need for a high level of quality assurance.

The objective of the current study was to introduce a low-cost inspection method for microfeatures, in both horizontal and vertical directions, which is suitable for microinjection molding parts. A small, round-shaped gap is created, referred to as a ‘checkpoint’ or ‘resistant point’, between the mold halves inside or outside the cavity. The filling of checkpoints reflects the actual behavior of the melt inside the mold. If the gap is filled with plastic, this indicates appropriate conditions for filling the part’s microfeatures. Also shown here is a two-point Go/No-Go gauge. One gap is designed to fill with material, confirming that the microfeatures are completely filled. The other gap is designed to remain empty, confirming that the mold remained securely closed during the injection process.

## 2. Design

**Figure 2:**
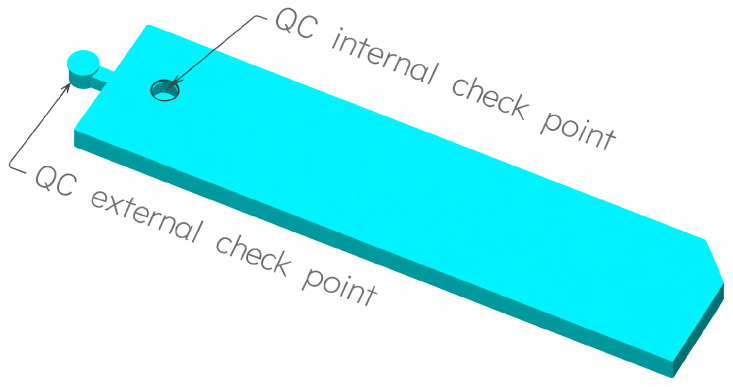
Diagnostic microfluidic body with internal and external QC checkpoints.

Checkpoints need to be correctly positioned; they should be placed close to the difficult-to-fill and/or critical features. If the checkpoint is going to cause disruption in functioning or disfigure the part, then it can be placed outside the part, as shown in Figure 2, and removed later.

**Figure 3:**
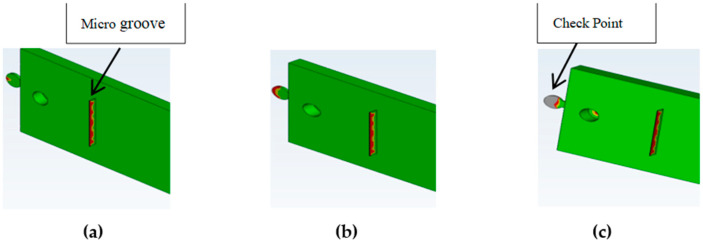
Filling simulation by Moldflow using checkpoint thicknesses. (**a**) 0.3 mm (no gray color and less red color), (**b**) 0.2 mm (more red color and no gray color), (**c**) 0.1 mm (more gray color) (gray color indicates no filling). In this simulation, mold temperature is 50 °C, injection pressure is 50 MPa, and melt temperature is 230 °C.

Computational Fluid Dynamics (CFD) simulation using Moldflow allows proper positioning of the checkpoints as well as determination of the required gap size (Figure 3). It is important that the checkpoint filling behavior is similar to microfeature filling; varying the checkpoint thickness will change its filling percentage, and if adjusted correctly, the filling will be similar to that of the microfeature. Simulation of a test part using Moldflow indicated that a 0.3 mm thick checkpoint was filled completely; in contrast, the 0.2 mm and 0.1 mm checkpoints are, respectively, partially and mostly unfilled. The checkpoint thickness is changed so that the filling of the checkpoint indicates the filling of the microfeature.

## 3. Experimental Setup

**Figure 4:**
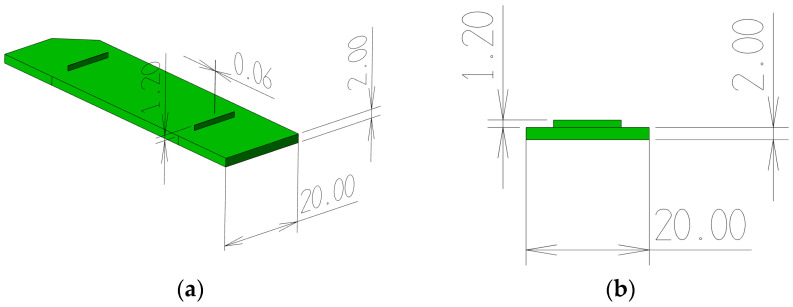
(**a**) A 3D model of the test microfluidic body (dimensions in mm). (**b**) Cross-section of the test chip with a 0.06 mm micro groove and a thickness of 2.0 mm.

Mold inserts with microfeatures were used to study the association between microfeature filling and checkpoint filling. The test part was a microfluidic body with features in the form of a microfeature, 10 mm long and 1.2 mm high, and having a thickness of 60 µm. The microfeatures were made with a draft angle of 0.88 degrees to aid the ejection of the part from the mold. The microfluidic body was 70 mm long and 20 mm wide, and had a thickness of 2 mm, as shown in Figure 4.

Four tooling cavity inserts were manufactured from tool steel (M461, Bohler, Germany). This material was selected for its high polishability, machinability, and strength (pre-hardened to 40 HRC). To form the microfeature in the mold, cylindrical inserts were made in two halves and machined to the required form. One half was fabricated by electric discharge machining (ARISTECH ZNC EDM, made in Taiwan), followed by polishing to a surface finish of 0.2 µm Ra, and then fastened together by a screw, which facilitated filling by avoiding any air traps, and also simplified the manufacturing process. The injection molding machine used in this study was a 50-ton (Engel e-victory 50, made in Austria) machine with an electrically driven screw, with a diameter of 22 mm.

**Figure 5:**
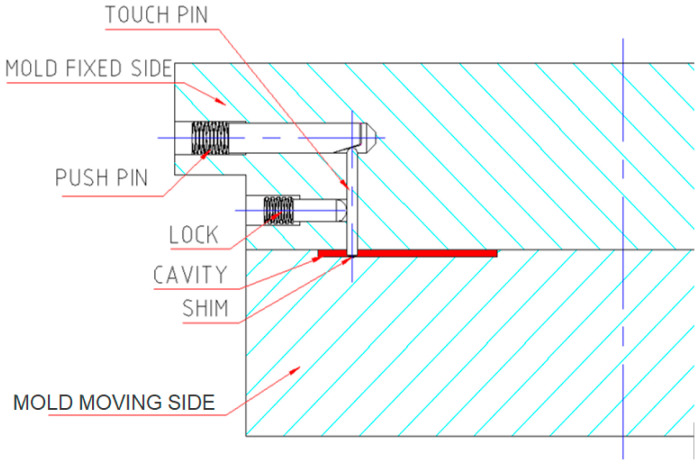
Parts of the checkpoint inside the mold.

An internal checkpoint on a chip with microfeatures was fabricated using a system that comprised four parts, as shown in Figure 5:

Push pin: pushes the touch pen by its taper end toward the cavity and the space shim. The push movement is made by a grip screw with an Allen key head.

Touch pen: It touches the space shim placed in the cavity.

Lock: It locks the touch pen to prevent it from moving forward to the punch side, and backward movement is prevented by the push pin. The grip screw provides the locking movement.

Spacer shims: These create the required gap between the touch pen and the cavity.

The mold must be designed to accommodate the above parts and to facilitate the setup operations.

## 4. Checkpoint Calibration Method

**Figure 6:**
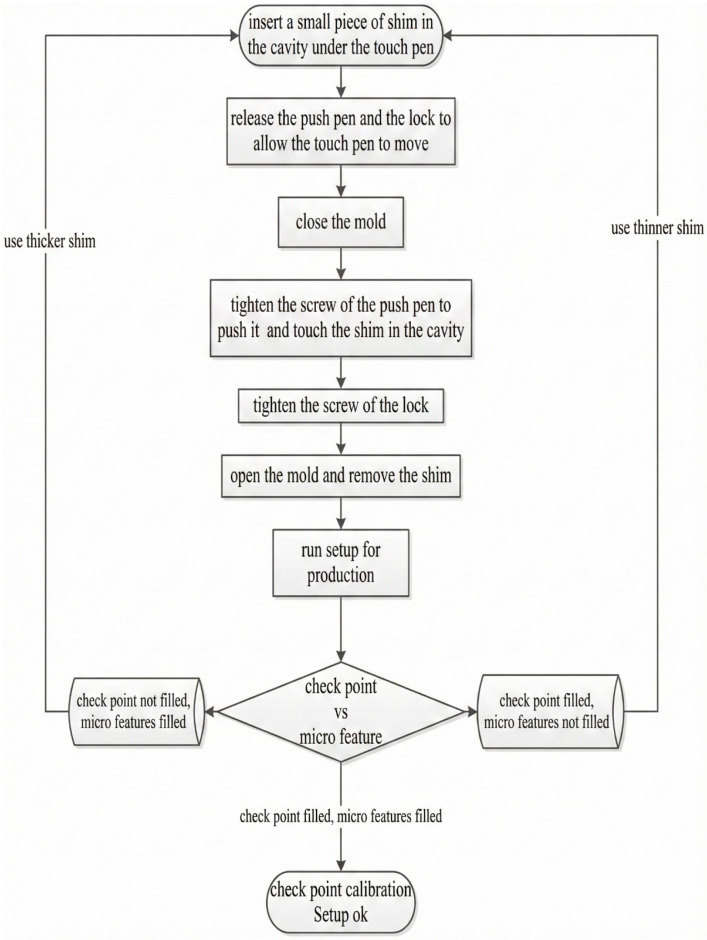
Checkpoint calibration flow chart.

In checkpoint calibration, steel shims of different sizes (10, 20, 30, 40, 60, 80, 100, and 120 µm) were used to set the gap between the touch pen and the cavity; the approach used for calibration is shown in Figure 6. Calibration is performed by creating the checkpoint and comparing the filling of the microfeatures with the filling of the checkpoint so that they are both filled at the same time. If the checkpoint is filled before the microfeature, the checkpoint gap should be reduced using a smaller shim. And if microfeatures are filled before the checkpoint, the checkpoint gap should be increased using a thicker shim. The filling can be evaluated by visual inspection; 8 iterations were typically performed.

## 5. Results and Discussion

Test parts were produced, and injection speed variations were used to control the filling of the micro groove and the checkpoint. Polypropylene (PP Buroge 470) with a melt flow index of 70 g/10 min was used for the experiments; the melt temperature was 270 °C. Plasticizing unit zones temperatures were 220 °C, 240 °C, 250 °C, and 270 °C; screw rotation speed was 100 PRM; the mold temperature was maintained at 30 °C; the specific injection pressure was 1350 bar; and the holding pressure was 50 bar. Injection time was 1.5 s, holding time was 2 s, and cooling time was 12 s. Checkpoint calibration was performed using different shim sizes. The gap in the checkpoint was chosen to be filled directly after the filling of the micro groove to ensure correct filling. The smallest steel shim size available was 10 µm, so this was used as the step size for calibration. The checkpoint gap was found to be 110 µm to achieve complete filling of the micro groove.

The experimental validation of microinjection molding simulations has been enhanced through multi-scale modeling approaches, providing better prediction accuracy for microstructured components [18]. Machine learning approaches have shown significant promise in the quality classification of injection-molded components, with studies demonstrating improved accuracy through quality indices and grading systems [19].

**Figure 7:**
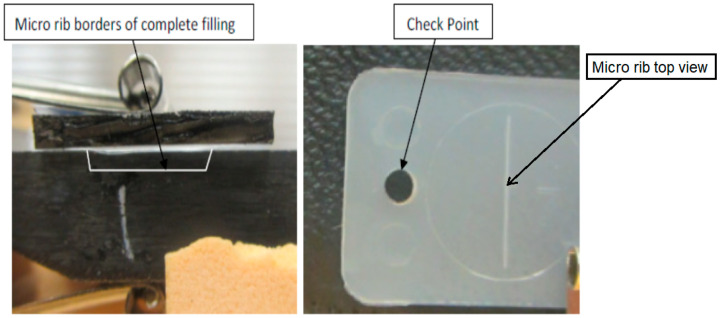
The checkpoint is not filled, and the groove is not filled (injection speed 7 cm^3^/s).

**Figure 8:**
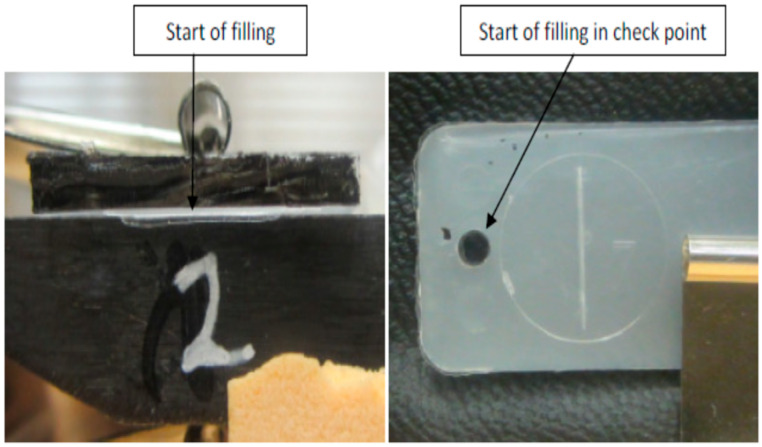
Onset of filling in the checkpoint and the groove (injection speed 16 cm^3^/s).

**Figure 9:**
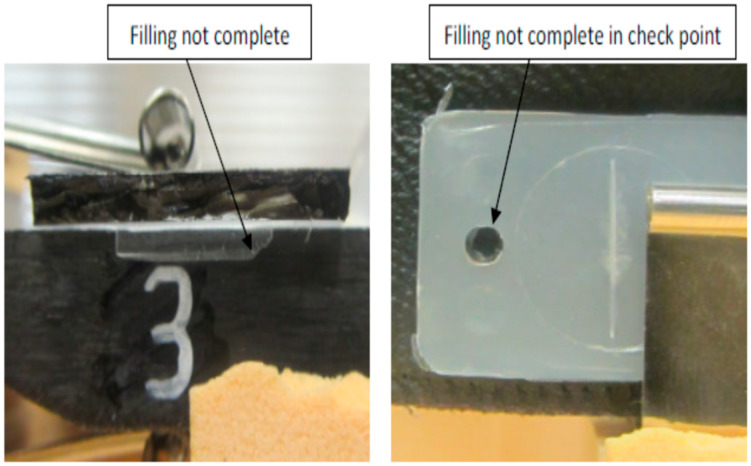
Checkpoint partially filled and groove partially filled (injection speed 60 cm^3^/s).

**Figure 10:**
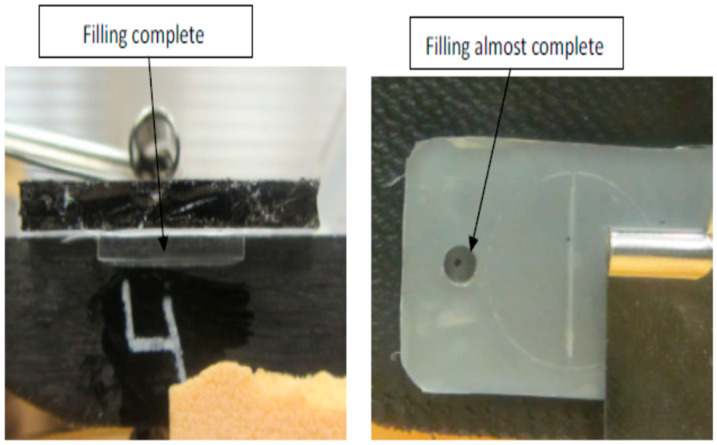
The checkpoint is almost filled, and the groove is filled (injection speed 96 cm^3^/s).

**Figure 11:**
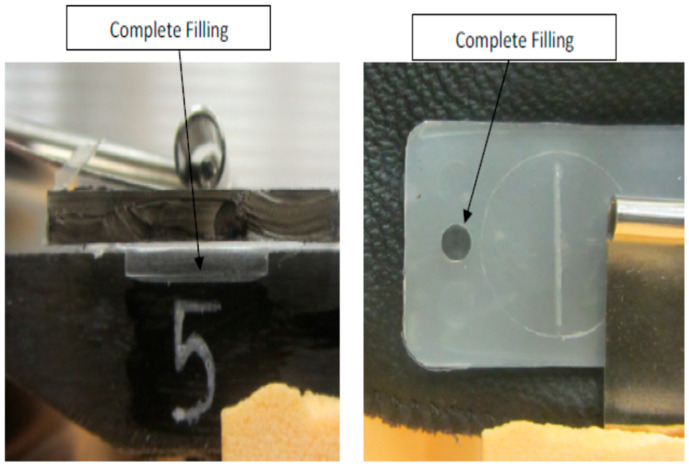
Checkpoint filled and the groove filled (Injection Speed 128 cm^3^/s).

Figure 7, Figure 8, Figure 9, Figure 10 and Figure 11 show the relation between the checkpoint filling and the microfeature filling by only changing the injection speed. An injection speed of 7 cm^3^/s was found to be too low to fill; both the checkpoint and microfeatures were not filled with plastic, as shown in Figure 7 (0% filling for both features). The start of filling of both the microfeature and checkpoint was observed at an injection speed of 16 cm^3^/s, as shown in Figure 8 (approximately 20% filling for both). Partial filling was observed at an injection speed of 60 cm^3^/s, as shown in Figure 9 (approximately 80% microfeature filling, 50% checkpoint filling). The microfeature was seen to be filled—with the checkpoint almost filled—at an injection speed of 96 cm^3^/s, as shown in Figure 10 (100% microfeature filling, 90% checkpoint filling), and both were filled at an injection speed of 127 cm^3^/s, as shown in Figure 11 (100% filling for both). The microfeature and checkpoint are located vertically and horizontally, respectively, on the microfluidic body. A correlation, therefore, exists between the filling of the two features, and this correlation can be used for controlling microfeatures regardless of the orientation of the features.

**Figure 12:**
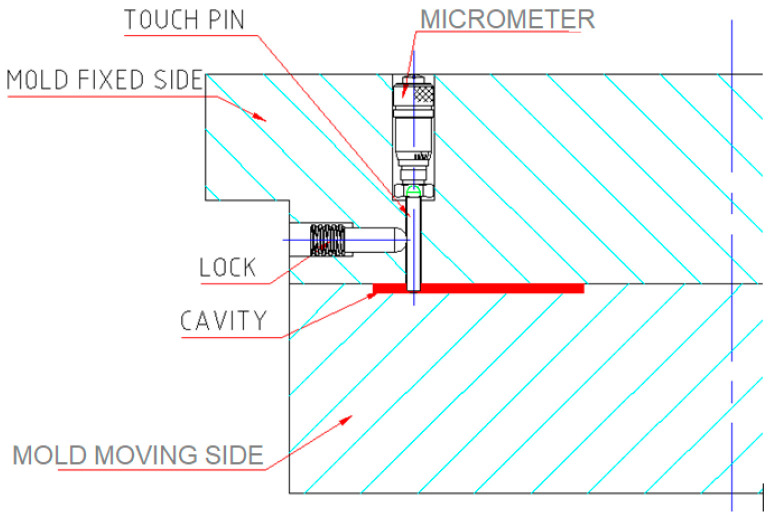
Mold equipped with a micrometer inside the cavity.

The size and accuracy of microfeatures that can be controlled by this technique depend on the smallest gap height that can be created in the checkpoint. If, for example, a custom-made micrometer having an accuracy of about 1 μm was placed inside the mold to replace the touch pen, the push pen, and the shims, then the accuracy of the proposed technique could be much higher (Figure 12).

The touch pen radius or micrometer pen radius divided by the checkpoint gap represents the aspect ratio of the checkpoint, and it can be related to the feature aspect ratio. Since the checkpoints are placed at the parting line of the mold, care should be taken to differentiate between checkpoint filling and the flash that occurs when molten material slips out of the part at the parting line. A dial indicator at the parting line of the mold halves will help to monitor the mold opening during mold filling and packing.

## 6. Go/No-Go Gauge Method

**Figure 13:**
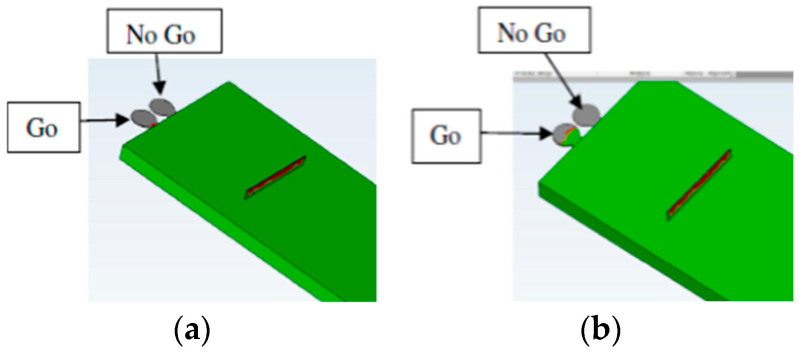
Moldflow simulation. Both gaps are not filled up.

To further ensure that this technique is reliable and to prevent an incorrect filling indication, a second checkpoint with a smaller gap is added to the same area. The second gap (checkpoint) should be open and not filled with plastic to indicate that there is no other reason for filling. If it is filled, this indicates that the mold opened during injection, and the filling of the second checkpoint is due to flash, as shown in Figure 13. To pass the parts through quality control, the first checkpoint should be filled with plastic, indicating that the filling is good enough to fill the checkpoint and the microfeatures, and the second checkpoint should remain unfilled with plastic, indicating that the mold did not open during the injection process and that the filling of the first checkpoint is not flash. Figure 13a shows the filling with a 0.1 mm gap No-Go checkpoint and a 0.2 mm gap Go checkpoint. Note that both gaps remained unfilled (gray color), whereas the micro groove was filled. Figure 13b shows the filling with a 0.1 mm gap No-Go checkpoint and a 0.25 mm gap Go checkpoint; the former is not filled, while the latter is half full.

**Figure 14:**
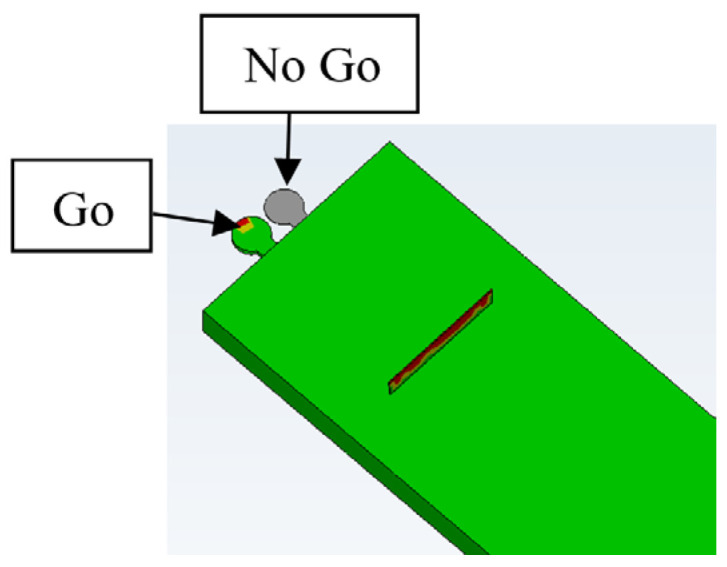
Moldflow simulation. The No-Go gap is not filled up, and the Go gap is full.

Figure 14 shows the filling with a 0.1 mm gap No-Go checkpoint and a 0.28 mm gap (Go) checkpoint. It can be seen that the No-Go gap is not filled, and the Go gap is full. This is the required indication for Go/No-Go. The No-Go checkpoint gap should not be 0 mm because the mold might open by, for example, 20 µm without filling it, which would give a wrong indication of the filling of the microfeatures.

The next simulation shows tests with different injection pressures and the relation between the filling of the microfeatures and the checkpoints. In the last test, the mold was loosely clamped, and it opened under pressure, causing the No-Go checkpoint to be partially filled.

**Figure 15:**
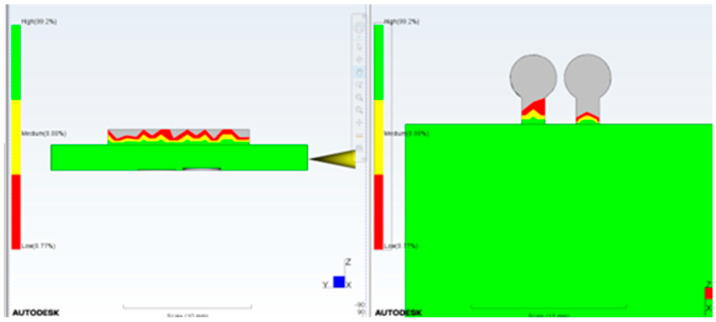
Injection pressure of 20 MPa. The Go gap is not filled, and the No-Go gap is not filled.

**Figure 16:**
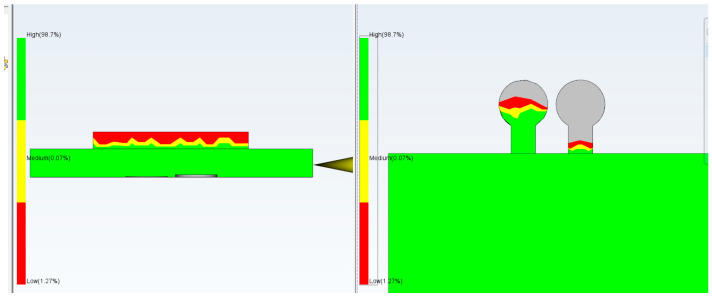
Injection pressure of 50 MPa. The Go gap is filled to 25% and the No-Go gap is not filled.

**Figure 17:**
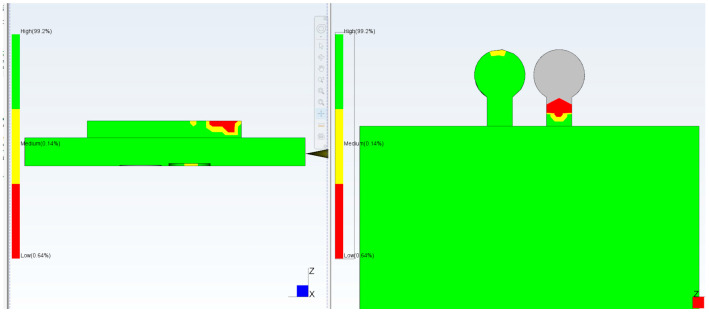
Injection pressure of 100 MPa. The Go gap is filled to 95% and the No-Go gap is not filled.

**Figure 18:**
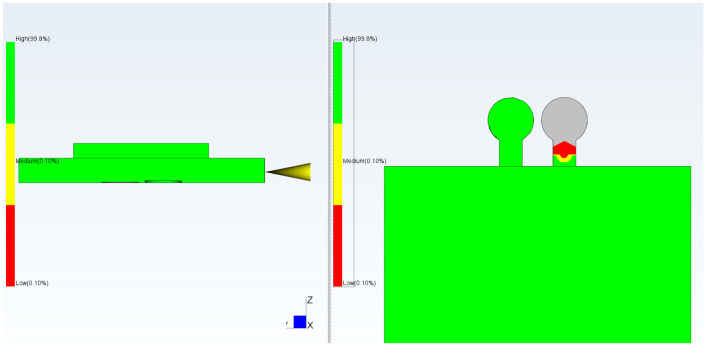
Injection pressure of 150 MPa. The Go gap is filled to 100% and the No-Go gap is not filled.

**Figure 19:**
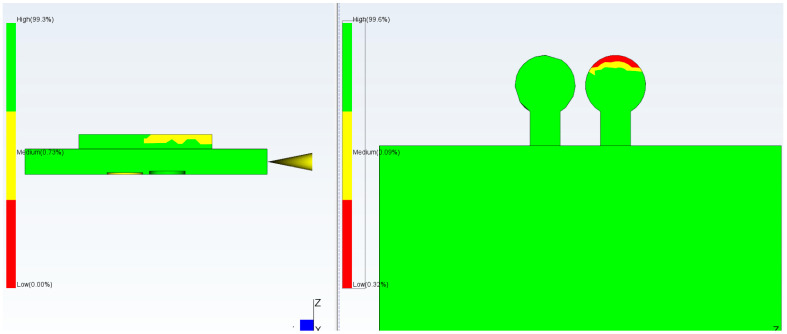
Injection pressure of 180 MPa. The Go gap is filled to 100% and the No-Go gap is filled to 80%.

Figure 15, Figure 16, Figure 17, Figure 18 and Figure 19 illustrate the relationship between injection pressure and the filling behavior of both the microfeature and the Go/No-Go checkpoints. As the injection pressure increases from 20 MPa to 180 MPa, the filling percentage of the microfeature and the Go checkpoint (180 µm gap) progressively increases, while the No-Go checkpoint (50 µm gap) remains unfilled until the pressure becomes excessive. At 150 MPa, the microfeature and Go checkpoint are fully filled, and the No-Go checkpoint remains empty, indicating ideal molding conditions. However, at 180 MPa, the No-Go checkpoint shows 80% filling, suggesting mold opening due to excessive pressure, which compromises part quality. These results validate the use of dual checkpoints as a reliable indicator of both filling completeness and mold integrity.

Table 1 summarizes the correlation between injection pressure and the filling behavior of microfeatures and checkpoints, along with the corresponding quality status of the molded parts. At low pressures (20–50 MPa), both the microfeature and the Go checkpoint are underfilled, leading to rejection due to short shots. At 100 MPa, although the microfeature is nearly filled (90%), the Go checkpoint is not filled, resulting in rejection for underfilling. The ideal condition is achieved at 150 MPa, where both the microfeature and the Go checkpoint are fully filled, and the No-Go checkpoint remains empty, indicating proper mold closure and complete filling. At 180 MPa, however, the No-Go checkpoint shows significant filling (80%), indicating mold opening under high pressure, which leads to partial rejection. This table underscores the effectiveness of the dual-checkpoint system in distinguishing between acceptable and defective parts based on injection pressure and filling behavior.

For validation, the No-Go checkpoint should not be filled whilst the Go checkpoint and microfeatures should be completely filled. This is to ensure that the dimensions of the microfeatures are within the required tolerances. Careful measurement using suitable equipment, like a coordinate measuring machine, stylus profile meter, scanning electron microscope (SEM), or optical microscope, can be used to ensure that the microfeature dimensions of the accepted parts conform to specifications. The successful correlation between checkpoint filling and micro groove filling demonstrates that this technique is directly applicable to the quality assurance of microfluidic devices.

The strong empirical correlation observed between the checkpoint filling and the microfeature filling is rooted in the fundamental physics of polymer flow in micro-scale geometries. The checkpoint is not merely a geometric proxy but a rheological equivalent of the critical microfeature. The calibration process, which determines the optimal checkpoint gap, is essentially a procedure to match the flow resistance or pressure drop (ΔP) of the checkpoint to that of the microfeature.

In microinjection molding, the flow is dominated by two critical phenomena:

High Shear Rate and Shear Thinning: Both the microfeature and the narrow checkpoint gap induce extremely high shear rates. The polymer’s viscosity is highly sensitive to this shear rate (shear-thinning behavior). By matching the geometry, we ensure that the melt flowing into both regions experiences a similar shear history.

Frozen Layer Development: The rapid cooling due to the high surface-area-to-volume ratio in microchannels leads to the swift formation of a solid frozen layer at the mold wall. This layer significantly reduces the effective flow channel thickness, drastically increasing flow resistance. The checkpoint gap is specifically tuned to replicate the rate and thickness of this frozen layer development relative to the flow length, ensuring that a short shot in the checkpoint mirrors any process variation leading to premature freezing in the microfeature.

This equivalence in flow resistance ensures that the checkpoint acts as a robust physical sensor, where its binary filling state is a direct consequence of the process conditions being sufficient to overcome the flow resistance of the most difficult-to-fill microfeature.

The general applicability of the checkpoint method extends beyond the specific polypropylene (PP) and micro groove geometry demonstrated here. The core methodology—calibrating the checkpoint gap to match the flow resistance of the critical feature—is universal. However, the calibration requirements change significantly with different materials and geometries. For high-viscosity engineering plastics (e.g., PEEK, high-molecular-weight PC), the higher melt viscosity and faster crystallization rates lead to a significantly greater pressure drop and more rapid frozen layer formation. Consequently, the checkpoint would require a larger gap to achieve the necessary flow resistance equivalence. Similarly, for high-aspect-ratio microfeatures (e.g., deep micro-wells), the increased risk of air entrapment and premature freezing means the checkpoint must be calibrated to a flow resistance that is highly sensitive to these failure modes. This may require a smaller, more precisely tuned gap.

Regarding the calibration process, the shim-based adjustment is a low-cost, practical solution for determining the optimal gap. While the initial search for the correct shim size is iterative and relies on visual inspection, the final, calibrated gap is defined by a precision-machined shim, ensuring high repeatability. The calibration is a one-time setup for a specific mold/material combination. Recalibration is only necessary if the material grade, the critical microfeature geometry, or the mold’s thermal properties are substantially changed. For high-volume, high-precision applications, the manual shim could be replaced by a micrometer-driven pin system for continuous, automated fine-tuning of the gap, although this would increase the system’s complexity and cost.

After calibration and validation, production can be started, and only the checkpoints are required to accept or reject parts. The inspection of the parts could be automated using optical sensors or even mechanical techniques. The external and internal checkpoints can be kept attached to the part as evidence for quality control procedures.

## 7. Integration with Smart Manufacturing and AI

The checkpoint method is a hardware-level innovation that is highly complementary to modern smart manufacturing and Industry 4.0 frameworks. Rather than being a standalone alternative to advanced QC, the checkpoint functions as a low-cost, physical-domain sensor that generates a high-value, binary ‘fill/no-fill’ signal for every injection cycle. This signal is an ideal input for adaptive control and data-driven quality systems. The binary output can be easily integrated into a digital twin of the molding process, serving as a real-time physical validation point for the virtual flow simulation. Furthermore, in a machine learning (ML) pipeline, the checkpoint’s signal provides a perfect, real-time ground truth label (good/bad part) for the most critical quality metric. This allows ML models to be trained to correlate complex, high-dimensional machine sensor data (e.g., nozzle pressure curves, screw position) with the actual microfeature filling outcome, enabling highly accurate, predictive, and adaptive process control. Thus, the checkpoint transforms the complex, analog problem of microfeature filling into a simple, digital signal that bridges the gap between physical process monitoring and intelligent, data-driven quality assurance.

## 8. Conclusions

A new approach has been demonstrated to inspect parts produced by injection molding. Checkpoints are used as a quality control tool for inspection in microinjection molding. The accuracy of the technique depends on the smallest gap height that can be created in the checkpoint (0.01 mm). The approach can be used as a Go/No-Go gauge to pass or reject the parts, and the checkpoint can be retained on the parts as evidence of validity. The approach allows simple and low-cost inspection that can be automated and is suitable for microinjection-molded parts. The checkpoint system presents a viable and robust QC solution for the growing field of microfluidic and lab-on-chip device manufacturing, where regulatory compliance demands traceable evidence of quality, and device functionality is critically dependent on the precise replication of internal microfeatures.

## Figures and Tables

**Table 1:** Relationship between injection pressure, microfeature filling, checkpoint filling, and part acceptance status.

	Injection Pressure MPa	Microfeature Fill %	Go Checkpoint (180 µm) Fill %	No-Go Checkpoint (50 µm) Fill %	Status Based on Checkpoints
1	20	10%	0%	0%	Reject (Short Shot)
2	50	25%	35%	0%	Reject (Short Shot)
3	100	90%	95%	0%	Reject (Under-filled)
4	150	100%	100%	0%	ACCEPT (Ideal)
5	180	80%	100%	80%	Reject (Mold Open)

## Data Availability

The original contributions presented in this study are included in the article. Further inquiries can be directed to the corresponding author.
